# A universal diagnosis syntax

**DOI:** 10.1186/s12911-023-02209-0

**Published:** 2023-07-31

**Authors:** Carl-Fredrik Bassøe

**Affiliations:** EkviMed AS, Solfjellveien 7, 1389 Heggedal, Norway

**Keywords:** Medical syntax, Clinical diagnosis, Clinical language

## Abstract

**Background:**

Diagnoses are crucial assets of clinical work and provide the foundation for treatment and follow up. They should be informative and customized to the patient’s problem. Common prefixes, morphemes, and suffixes may aid the implementation of expressions that generate diagnoses.

**Results:**

Apt choices of symbols plays a major role in science. In this study, the variables e, o, and p are assigned to names of an etiological agent, a disorder, and a pathogenetic mechanism, respectively. The suffix -itis designates infections, allergies, inflammation, and/or immune reactions. Diagnoses (d) are generated by the formula d:= e&o&p where ‘&’ means concatenation and ‘:= ’ means assignment. Thus, with e:= ’*Staphylococcus aureus* ‘, o:= ’endocard’, and p:= ’itis’, d:= e&o&p generates the diagnosis d = ’*Staphylococcus aureus* endocarditis’. Diagnoses formed this way comply with common clinical diagnoses. Certain extensions generate complete, systematic medical diagnoses that are applicable to all medical specialties. For example, common medical prefixes, morphemes, and suffixes give rise to o = ’hypothyroidism’, o = ’tachycardia’, and o = ’hypophagocytosis’. The formula scales well with the developments in clinical medicine, systems biology, molecular biology, and microbiology. The diagnosis generating formula d:= e&o&p requires meticulous analysis of the components of diagnoses plus the introduction of appropriate variables and terms. Terms partition on established clinical categories and adhere to established clinical nomenclature. The syntax generates universal medical diagnoses.

**Conclusions:**

The present study concerns a universal diagnosis syntax (UDS) that generates diagnoses using the formula d:= e&o&p with several extensions described in the study. The formula is easy to learn and covers diagnoses in all medical specialties. The present work succeeded in creating diagnoses from the formula. The fundamental insight is that no matter how complicated a diagnosis is it can be generated by a systematic process, which adds terms one by one. UDS may have implications for medical education and classifications. The formula lays a foundation for structured clinical decision-making. Formulas are hallmarks of hard science. So, d:= e&o&p anticipates a scientific medical revolution.

## Background

It is hard to state the rules that govern diagnoses. Some diagnoses are ambiguous. Other seemingly incoherent strings of words are understandable utterances. In addition, there is a wide variety of diagnoses. The aim of this work are diagnoses that are useful and allow perfectly understandable communication.

Current medical diagnoses are a blend of names of diseases, disorders, syndromes, and clinical findings. The names of diseases, disorders and syndromes consist of proper names and rigid descriptions of clinical entities. Proper names such as *schizophrenia* and *mononucleosis* are invented and *Conn syndrome* and *Hirschsprung’s disease* are surnames. Such diagnoses cannot be constructed from morphemes that refer to the etiology, disorder or pathogenetic mechanisms. For this reason, proper names do not count as diagnoses in the present sense of the word.

Systematic diagnoses such as *bacterial sinusitis*, *E. coli cystitis* and *myocardial infarction* are considered to be rigid (definite) descriptions. [1] All these diagnoses may refer to an etiology, one or more disorders and/or pathogenetic mechanisms. In this work, only rigid descriptions count as medical diagnoses. We also require a rather stringent but informal syntax that underlies informal systematic diagnoses such as *Acute Neisseria* *meningitidis meningitis*, *bacterial tonsillitis*, *Streptococcal tonsillitis*, *chemical alveolitis*, *hereditary spherocytosis* and *idiopathic pulmonary fibrosis* that are obtained from standard medical textbooks and classifications.

The same name is encountered in several diagnoses, but the names may refer to different objects and events. For example, β-cells are found both in the pancreas and pituitary gland. Also, the morpheme *itis* may point to inflammation, infections, allergies, and autoimmune reactions. This study aims to resolve ambiguities that arise from different meanings of terms in different contexts.

The history of medicine tells a long tale of misnomers, for example the obsolete diagnosis *pachymeningitis hemorrhagica interna* [2], p. 32]. The diagnoses *hysteria* and *neurasthenia* were common about a century ago but are rarely used today. Medicine has the capacity of clearing away misnomers, but some still remain. Furthermore, diagnostic terms demand a clear morphology. For example, the term *itiscys* is an incomprehensible misnomer, whereas *cystitis* is a well-formed expression that physicians immediately understand. The diagnoses generated in this study disallows misnomers.

Clinical findings (symptoms, signs, and supplementary investigations) are part of arguments used in clinical decision-making (CDM) to select and create diagnosis. For example, *dysphagia* and *dyspepsia* refer to unclear collections of clinical findings. The present work separates arguments from diagnostic conclusions (diagnoses) and does not allow collections of clinical findings as diagnoses. By doing this we can rid diagnoses of misnomers caused by mixing etiology, disorder and pathogenetic mechanisms with clinical findings.

At least 47 distinct ways for expressing *myocardial infarction* appear in clinical notes [3]. Because of the variety of such diagnoses, they deserve the label natural medical language. This article concerns the development of a universal diagnosis syntax (UDS) that standardizes diagnoses and assigns only one composite form to each diagnosis.

Logic, mathematics, and chemistry have profited from symbolic notations ([4], p. 13). Morphology and syntax fall within the domains of linguistics, logic, the philosophy of language and informatics ([1, 5] p, 32–3, 6, 7). An appropriate syntax for a formal language requires a vocabulary and a set of rules ([6] p.6–7). Transformational-generative grammars have proved useful for language production ([7, 8] p.21). Generative grammar evolved into formal language theory (FLT), which has a wide range of applications [9]. But no such symbol system is available to clinical medicine. This study introduces symbols and a formula that generates systematic medical diagnoses.

Widely used medical classifications such as the 10^th^ International Classification of Diseases (ICD-10) [10] and the 2^nd^ International Classification of Primary Care (ICPC-2) [11] essentially list proper names and descriptions of diagnoses. They are used in Electronic Patient Records (EPR) to select diagnoses and their associated codes. Any such classification lack important diagnoses. The lists last for some years. Extending and revising them is difficult and time-consuming, and backwards compatibility remains a serious problem.

Static classifications seem to be unsuited for the changing world of CDM. Combinatorial classifications such as the Systematic NOmenclature in MEDicine (SNOMED) may overcome these problems [12]. They account for novel diseases and syndromes simply by adding new elements to existing lists and allow new elements to combine with existing elements. However, SNOMED is secluded from the public and the classification lacks an underlying clinical model. Since SNOMED’s syntax has no semantics the classification cannot be validated.

Medical terminologies have advanced significantly in the last years ([13] p.124–35) but how to reduce the variety of disease definitions remains an important unsolved problem ([14] p.1, 16). Lack of an agreed infrastructure for terminology is identified as one of the major barriers to information interchange and integrate EPR with medical knowledge bases.

The Unified Medical Language System (UMLS) is an advanced effort towards the integration of biomedical terminologies [15, 16]. The Semantic Network, a component of the UMLS, is a structured description of core biomedical knowledge consisting of well-defined semantic types and relationships between them [17]. However, UMLS does not provide sufficient logic-based structures [18]. Concept-oriented and logic-based approaches are beneficial for creating categorical terminological structures.

The Generalized Architecture for Language Encyclopaedias and Nomenclatures in Medicine (GALEN) and UMLS are large thesauri [18]. One aim of these projects is to bridge the gap between different terminology systems using a conceptual model and mapping facilities to natural language expressions and coding schemas [17, 19, 20].

The GALEN project aims to bridge the gap between different terminology systems through a terminology server, which contains a conceptual model and mapping facilities to natural language expressions and coding schemas [19, 20]. Several projects have been launched to converge clinical terminologies towards a grand unified system [21, 22]. The complexity and high number of medical and health terminologies lead more recent projects to limit their attack on man–machine and machine-machine interoperability to limited domains [23–27]. Despite ongoing work toward shared data formats and linked identifiers, significant problems persist in semantic data integration across heterogeneous biomedical data sources [28]. It remains difficult to establish shared identity and shared meaning.

A formal language is characterized by its vocabulary and syntax ([1], p.26). The vocabulary consists of three basic expressions: logical terms, logical variables, and auxiliary signs such as brackets. There is also a set of rules which show how expressions can be combined to make new expressions. The meaning of expressions is determined by Frege’s principle of compositionality: the meaning of a composite expression is wholly determined by the meanings of its component parts and the syntactic rule by which it was formed [1, 4]. There are opponents to this view ([29] p. 219–37) but for diagnoses we adhere to Frege’s principle.

Systematic diagnoses can be decomposed into their component parts [30]. For example, *bacterial conjunctivitis* can be parsed into the etiology *bacteria*, the body part *conjunctiva* and the pathogenesis *itis*. Finally, a flexible combinatorial classification, which was based on the same components was implemented in the early days of EPR in Norway [31] and worked according to purpose in another EPR ([32, 33] p.212–4).

We tried a dynamic approach that lets physicians write ordinary textbook diagnoses into diagnosis fields in an EPR [34]. The diagnoses were some years later associated with an ICPC-2 code. Also, physicians could change the diagnosis name associated with a code when the name was inappropriate. That this option was used shows the advantage of being able to change diagnoses.

Various clinical specialties use the same diagnoses and operate within the domains etiology, disorders, and pathogeneses. This indicates that all specialties may use a common formula for generating diagnoses.

This study aims to base a universal diagnosis syntax (UDS) on a formula and clinically meaningful terms. The assignment of strings to variables is purely syntactic and the strings do not embody meaning by themselves [9]. The variables of the formula are instantiated with names of concepts that are derived from an empirical clinical model [35]. The relationship between the present syntax and its semantics is investigated separately.

Health personnel that are not acquainted with formal languages or informatics need not worry. All the rules and terms are explained and aligned with standard school medicine. Use and mention of terms are distinguished typographically. Use: Lungs are in the chest. Mention: *Lungs* consists of five letters. Terms mentioned in formula are embraced by single brackets. Thus, in a formula *lung* is converted to ‘lung’. Also, *Lungs* is a string of characters in [a-z, A-Z]. Rules for generating diagnoses show how to construct them and how to test them and show whether they are valid or not.

## Methods

This study aims at a syntax for systematic medical diagnoses. The definition of the syntax derives from the structure of definitions in first order logic [1]. The definition consists of a basic vocabulary and a formula for generating diagnoses. The formula is later extended to diagnoses in all medical specialties.

The design of the vocabulary and formula is iterative and evolved over many years. The vocabulary is derived from medical textbooks, reviews, and numerous searches on PubMed.

The setting is university studies (M.D., Master of Information Technology, and Master of Philosophy), practical work (surgery, primary care, occupational medicine, pathology, oncology), awarded specialties (general internal medicine and hematology), research experience (experimental medicine (PhD), medical informatics (PhD), and clinical research), and teaching as professor (internal medicine and health informatics).

The study involves no participants other than the author, and no drugs, other materials, processes, or statistical analysis.

## Results

### Universal diagnosis syntax (UDS)

Systematic diagnoses are character strings that name elements within the clinical domains etiology, disorders, and pathogenesis. UDS constitutes terms, variables, and rules that together generate medical diagnoses. That the character string ‘abc’ is assigned to the variable v is symbolized by v: = ’abc’ where := is the assignment symbol. The equality sign = and the inequality sign ≠ are used to check whether two-character strings are identical or not, or whether two variables refer to the same character string or not.

Concatenation joins character strings end-to-end. The symbol for concatenation is &. For example, the strings ‘abc’ and ‘abc’ are concatenated by ‘abc’ & ‘ abc’ into ‘abcabc’ and ‘abc ’ & ‘abc’ = ‘abc abc’ since the left hand ‘abc ‘ ends with a space. Since ‘abc’ = ‘abc’ whereas ‘abc’ ≠ ‘acb’ concatenation is not commutative. If v_1_: = ’abc’, v_2_: = ’abc’, and v_3_: = ’acb’ then v_1_ = v_2_, v_2_ ≠ v_3,_ and v_1_ ≠ v_3_.

### Definition 1

i) The basic vocabulary of UDS consists of terms, connectives, and variables.
ii) UDS connectives are *:*= , &, = and ≠ . & takes precedence over := .
iii) Terms are strings of characters such as *bacteria* and *kidney*, which refer to the objects bacteria and kidney, respectively.
iv) Terms obtain from the underlying sets of the etiology (E), disorder (D) and pathogenetic mechanisms (P), which derive from clinical model.
v) The variables e, o, and p are assigned to terms from E, O and P, respectively. Thus, in e : = ’bacteria’ the string ‘bacteria’ from E is assigned to the etiology variable e. Thus, E, O, and P are different types.
vi) The variables restrict the types of terms that can be assigned to them. The left hand side of the connective := holds one and only one variable and no terms and no other connectives. The right hand side of := contains one term or a concatenation of an arbitrary number of variables and terms. Alternative terms that can be assigned to one variable are discriminated by the sign |.
vii) Diagnoses are generated by
  1 $$d:= e \& o \& p$$
viii) Formula 1) defines the order of the variables uniquely from left to right. The variables are non-commutative, which means that d:= e&o&p is a valid formula, but d:= e&p&o, d:= o&e&p, d:= o&p&e, d:= p&e&o, and d:= p&o&e are invalid. In other variables the first letter is uppercase letter followed by lower case.
ix) Only strings generated by i) to vii) in a finite number of steps are diagnoses.

In order to increase readability ‘&*’* associated with variables can be discarded whereby e&o&p is abbreviated to eop.

The variables e, o and p partition diagnostic terms into the three categories etiology, disorder and pathogenesis, respectively. In contrast to first order logic, the categorical distribution of descriptive terms such as *streptococcal*, *tonsil* and *fibrosis* are important to UDS diagnoses ([1], p.7). This empirical lexical prerequisite has no exceptions.

The following pseudocode generates diagnosis in UDS that accord with formula 1:

$$\frac{\!\!\!\!\!\!\!\!\!\!\!\!\!\!\!\begin{array}{l}\mathrm{e}:=\ ' \mathrm{ E.coli}' \\ \mathrm{o}:= \ '\mathrm{cyst}'\ \\\mathrm{p}:=\ '\mathrm{itis}'\\\mathrm{d}:=\mathrm{eop}\end{array}}{\mathrm{d}=\ '\mathrm E.coli\mathrm\ {cystitis}'}$$

Clearly, the same diagnosis is generated independently of the sequence of instantiation of e, o, and p. Therefore, formula 1) lends the same structure to all UDS diagnoses. All such diagnoses are well-formed formulas. All the variables in formula 1) are important for treatment and follow up. Definitions i-ix are assumed to hold for all clinical specialties.

Additional rules discriminate common types of diagnoses:

1. **Diseases** are complete and systematic diagnoses generated by the formula d:= eop and requires that e ≠ ’’, o ≠ ’’, and p ≠ ’’. For example, infections refer to the triple of microorganism, disorder, and immune reaction. Therefore, infections are diseases. By definition, hereditary disorders are diseases.
2. **Disorders** are incomplete systematic diagnoses generated by d:= o.
3. **Syndromes** are incomplete systematic diagnoses generated by d:= op. In UDS syndromes are not names of genetic disorders consisting of collections of clinical findings.
4. Diagnoses with unknown anatomical localization are generated by d:= ep or d:= p.
5. Etiological agents alone do not give rise to diagnoses. Hence, d:= e is not a valid diagnosis. Etiological agents that are not translocated into a primary affected body part only qualify as risk factors.

Formula 1) is implemented from an algorithm and the knowledgebase contains complete lists of terms.

### Body parts—scope

Diagnoses often describe the localization of a disorder or pathogenetic mechanism such as *bilateral crural edema* and *finger eczema*. Many dermatological disorders such as psoriasis are characterized by their regional distribution. The anatomical scope of such disorders is limited.

The scope of disorders is described by terms that name body parts. Scope partitions into segments *Seg* and regions *Reg*. The site variable *Site* determines absolute and relative locations. The ‘right kidney’ is an absolute reference. In contrast, in ‘tumor to the left of the right kidney’ the tumor is located relative to the right kidney. Recursive use of *Site* is allowed. Thus, Site := ‘ above’, can be followed by Site := Site & ‘ left kidney’. The vertical bar | discriminates alternatives. Typical examples are:

Seg := ‘C1’ | ‘C2’ | ‘C3’ | ‘C4’ | ….

Reg := ‘ frontal’ | ‘occipital’ | … | ‘ abdominal’ | ‘crural’ | ….

Site := ‘right’ | ‘left’ | ‘bilateral’ | ‘in’ | ‘left of’ | ‘to the right of’ | ‘to the left of’ | ‘to the right of’ | ‘lateral to’ | ‘medial to’ | ‘proximal to’ | …

The scope is considered by modifying formula 1) into

2 $$d:=\mathrm{Seg }\&\mathrm{ Reg }\&\mathrm{ Site }\&\mathrm{ eop}$$

In case of *crural edema* Reg := ’crural ’ and p := ’edema’. The etiology has not been investigated and is empty, i.e., the initialization leaves Seg = Site = e = o = ’’. Therefore, formula 2) leads to d:= ’crural edema’, which is obviously an incomplete diagnosis. Note that *crural swelling* is a sign, but *crural edema* requires a clinical inference to count as a pathogenetic mechanism.

The variable o incorporates morphemes of names of body parts held by the variable N. The variable N_X_, holds the morpheme of an organ system, organ, organ part, tissue, or a cell. The index X is instantiated by P, T and S, which discriminate the variables that hold names of parenchyma N_P_, tubes N_T_ and slits|cavities N_S_, respectively. Thus,

N_P _:= ‘hepat’ | ‘nephr’ | ‘dermat’ | ‘bone’ | ‘tendon’ | ‘myocard’ | ‘ligament’ | ‘intervertebral disc’ | ‘meniscus’ | ‘cornea’ | ‘lens’ | ‘corpus vitreum’ ….

N_T _:= ‘Eustachian tube’ | ‘coledoch’ | ‘salping’ | ‘arterio’ | ‘aortic ‘….

N_S _:= ‘cholecyst’ | ‘pyelo’ | ….

The tissue N_I_, cell line N_L_, cell variable N_C_ and their terms are:

N_I _:= ‘squamous cell’ | ‘lymphoid’ | ‘connective tissue’ | ‘adipose tissue’ |’osteo’ |’fibro’ |’lipo’ | ….

N_L _:= ‘erythropoiesis’ | ‘spermatogenesis’ | ….

N_C _:= ‘β-cell’ | ‘α-cell’ | ‘B-lymphocyte’ | ‘parietal cell’ |’adipocyte’ |’fibroblast’ |’mast cell’ |’neuron’ |’purkinje cell’ | ‘striated muscle cell’ | ‘basal cell ‘ ….

The subcellular level cover subtler disorders. In the variable N_CY_ the index C points to cells and Y to morphemes of organelles and other subcellular structures. The variables and their corresponding terms are:

N_YO _:= ‘dendrite’ | ‘perikaryon’ | ‘axon’ | ‘synapse’ | ‘cytoskeleton’ | 

‘microtubule’ | ‘mitochondria’ | ‘Golgi apparatus’ | ‘nuclear double membrane space’ | ‘cell nucleus’ | ‘vacuole’ | ‘lysosome’ | ‘endosome’ | ‘phagosome’ | ‘sarcoplasmic reticulum’ | ….

The variables can be concatenated into N := N_P_N_I_N_L_N_C_N_O_, which allows all possible combinations of morphological disorders in subcellular structures of cells in a tissue residing in an organ.

Some diagnoses generated with the variables N_P_, N_T_ and N_S_ are concatenated with suffixes like ’ism’, and ‘itis’. For example, ‘gonad’ & ‘ism’ becomes ‘gonadism’, ‘thyroid’ & ’ism’ is ’thyroidism’ and’thyroid’ & ‘itis’ contracts to ‘thyroiditis’. The formula secures that suffixes correspond with the actual etiology, disorder, and pathogenesis.

### Direction of change—Dir

Morphological and functional directions of change are denoted by well-known clinical prefixes. The direction may differ in the etiology, disorder, and pathogenesis. Also, morphology and function may require different directions of change even in one and the same diagnosis. The direction of change is assigned to the variable Dir_X_^Y^. Dir_E_, Dir_O_^Y^, Dir_T_^Y^, Dir_S_^Y^ and Dir_P_ are used with Dir_P_^Y^ the etiology E, and parenchyma O, tube T, or slit S disorder, and pathogenesis P, respectively. For the etiology and pathogenesis the superscript Y is ignored as in Dir_E_ and Dir_P_.

Dir_X_^M^ and Dir_X_^F^ discriminate morphology M from function F. Some other X and Y values are set aside for specific parenchyma functions and pathogenetic mechanisms. For example, *hypoglycemia due to hyperinsulinemia* belongs to the pathogenesis and requires X := ’P’. In this context, Y = B and Y = H refer to metabolites and regulators, respectively. Therefore, Dir_O_^B ^:= ’hypo’ and Dir_O_^H ^:= ’hyper’ depend on context. Accordingly, the scope of Dir_X_^Y^ is limited to the term it associates with. The direction of change is assigned to the variable Dir_X_^Y^ as follows:

Dir_X_^Y ^:= 'a' | 'an' | 'hypo' | 'normo' | 'hyper' | 'meta' | 'dys' | 'neo' | ‘extra’ | 'brady' | 'tachy' | 'localized' | 'generalized' | ‘paroxystic’ | ‘seizure’ | ‘epi’ | ‘allo’ | ‘anti’ | ‘iso’ | ‘ deficiency’ | ‘para’ | ‘elevated ‘ | ‘diminished ‘ | …

These prefixes pertain to the disorder variable o in formula 1) and 2) and describe the direction altered morphology and/or function. Crude disorders of function are generated by O_P_^F ^:= Dir_O_^F^&N_P_. The variable F_T_ is a tube function. Diagnoses of tube functions are generated by O_T_^F ^:= N_T_&Dir_T_^F^&F_T_. Thus, if N_T _:= ’arterial ‘, Dir_T_^F ^:= ’hypo’ and F_T _:= ‘tension ‘ then the diagnosis is *arterial hypotension*. If a term starts with a vocal then ‘a’ is automatically replaced with ‘an’. The terms associated with Dir_X_^Y^ are always selected from the list above, which is implemented in the knowledge base.

### The disorder variable o

The diagnosis *Staphylococcus aureus myocarditis* does not tell whether the infestation causes heart failure, arrhythmia or has no adverse effect on heart function. In fact, the diagnosis is incomplete with regard to heart morphology and function. Complete diagnoses require variables and terms that inform on the morphology and function of the affected body part.

The variable O_X_^Y^ additionally discriminates parts of the general organ. X = P, X = T, X = S, X:= I, X:= L, X:= C and X:= O refer to parenchyma, tubes, slits|cavities, tissues, cell lines, cells, and cell organelles, respectively. Thus, diagnoses of morphology and function in particular body parts are given by

3 $$O_X^Y:=N_XS_V{KLDir}_X^YY_X^Z$$

where Y = M and Y = F means morphology and function, respectively. S_V_ represents the suffixes S_E_, S_O_ or S_P_ that can be attached to N_X_, for example S_P _:= ’lithiasis’ attaches to N_O_ = ’nephro’ in *nephrolithiasis*.

The variables K and L only concern names of metabolites and regulators. Otherwise, K := ’’, L := ’’ and 3) simplifies to O_X_^Y ^:= N_X_&Dir_X_^Y^&Y_X_^Z^. The index X pertains to the body parts as described above. The superscript Z handles specific functions and holds a single character (see below). In morphological diagnoses Z := ’’ and formula 3) simplifies to O_X_^M ^:= N_X_&Dir_X_^M^&M_X_ and O_X_^F ^:= N_X_&K&L&Dir_X_^F^&F_X_^Z^, respectively. For example, disorders of parenchyma morphology are generated by O_P_^M^ := N_P_&Dir_X_^M^&M_P_.

The lungs, liver and kidneys are composite organs that consist of several parenchyma, tube systems, and slits/cavities. Disorders in composite organs may concern more than one part of the organ. This applies to many liver diseases accompanied by hepatocyte swelling that give rise to both intrahepatic cholestasis and loss of liver functions. All these changes should be explicitly described. Very complex disorders can be generated by

4 $$\begin{array}{cc}{O}^{M}:=& {O}_{P}^{M}{O}_{T}^{M}{O}_{S}^{M}{O}_{I}^{M}{O}_{L}^{M}{O}_{C}^{M}{O}_{O}^{M}\\ {O}^{F}:=& {O}_{P}^{F}{O}_{T}^{F}{O}_{S}^{F}{O}_{I}^{F}{O}_{L}^{F}{O}_{C}^{F}{O}_{O}^{F}\end{array}$$

In most clinical situations only one variable is instantiated, and the others remain empty. For example, common disorders such as *myocardial hypertrophy* and *arterial hypertension* are generated by O^M ^:= O_P_^M^ (with N_P _:= ’myocard’, S_O _:= ‘ial ’, Dir_O_^M ^:= ’hyper’, M_P _:= ’trophy ’) & ' and ' & O^F ^:= O_T_^F^ (with N_T _:= ’arter’, S_T _:= ‘ial ’, Dir_T_^F ^:= ’hyper’, F_T _:= ’tension’) respectively, and keeping the other variables in formula 4) empty.

Dir_X_^Y^ can be considered as a function in itself. Parentheses are included in UDS to limit the scope of Dir_X_^Y^. Therefore, Dir_X_^Y^() may range over more than one morphology and function. For example, Dir_O_^M^(M_P_F_P_) = Dir_O_^M^&M_P_&Dir_O_^F^&F_P_. Thus, if Dir_O_^M^ = ’hypo’ then Dir_O_^M^(M_P_F_P_) = ’hypo’&M_P_&’hypo’&F_P_.

The direction of morphological and functional disorders of one body part do not always correlate. For example, goiter and hypothyroidism are characteristic of iodine deficiency. Another example is hepatocellular carcinoma with hepatic hypofunction. Hence, accurate diagnoses may need descriptions of both morphology and function. The variable o in formula 1) and 2) holds the variable O^Y^ that pertains to morphology if Y = M and function if Y = F. This opens for the possibility to name causal relations. The diagnoses of compound disorders are given by

5 $$o:=O^M{\And}^{\prime}\text{causing }^{\prime}{\And}O^F$$

A typical example is o = ‘hepatic carcinoma causing hepatic hypofunction’.

#### Parenchyma and connective tissue

##### Morphology

Morphological parenchyma diagnoses stored in O_P_^M^ are generated by X := P, Z = ’’ and formula 3). Since Y = M the following variables hold morphological terms:

N_P_:=M_I_:=M_C_:= 'tumor’ | ‘adenoma’ | ‘oma’ | ‘pathy’ |’trophy’ | ‘plasia’ | ‘sarcoma’ | ‘carcinoma’ | ‘adenocarcinoma ‘ | ‘papillary carcinoma ‘ | ‘fissure ‘ | ‘fracture ‘ | ‘genesis’ | ‘degeneration’ | ‘ porosis‘….

In the unspecific diagnoses all Dir_O_^Y ^:= ‘’. Thus, if N_P _:= ‘thyroid ‘ and M_P _:= ‘tumor’ then formula 3) gives O_P_^M^ = ‘thyroid tumor’. Alternatively, if N_P _:= ‘thyroid ‘ and M_P _:= ‘adenoma’ then O_P_^M^ = ‘thyroid adenoma’. More serious tumor disorders are given by Dir_O_^M ^:= ‘neo’ and M_P _:= ‘plasia’, where for example N_P _:= ‘thyroid ‘ leads to O_P_^M^ = ‘thyroid neoplasia’. Unknown morphology, i.e., O_P_^M ^:= ’’ is also allowed.

Certain benign morphological tissue diagnoses are generated by 3) with X := I and M_I _:= ‘oma’. Then N_I _:= ‘lip ‘ gives O_I_^M^ = ‘lipoma’ and if N_I _:= ‘fibr ‘ we get O_I_^M^ = ‘fibroma’. These simple concatenations cover a wide variety of benign tumors. In contrast, if N_I _:= ‘lymph ‘ we get O_I_^M^ = ‘lymphoma’. The latter covers tumors with varying degrees of malignancy, and it is necessary to specify the degree of the malignancy (see below). M_I _:= ‘sarcoma’ allows the generation of ‘liposarcoma’, ‘fibrosarcoma’ and ‘rhabdomyosarcoma’. The concatenation with region or site generates the anatomic localization of other *omas* and *sarcomas* in accordance with the anatomic scope of the disorder.

The tissue and cell type are generated by simple rules. If N_I _:= ’squamous ’ and Dir_O_^M ^:= ‘neo’ then M_I_ = ‘carcinoma’. In this case and with N_P _:= ‘pulmonary ‘ formula 3) gives O_P_^M^ = ‘pulmonary carcinoma’. Likewise, if instead N_P _:= ‘gastric ‘ we get O_P_^M^ = ‘gastric carcinoma’.

The brain is often considered to be something very special and complex. However, morphological brain disorders behave as other organs and tissues. If N_C _:= ‘astrocyt ‘ and M_P _:= ‘oma’ then formula 3) gives O_P_^M^ = ‘astrocytoma’. ‘anaplastic neuroblastoma’ is generated by the same principle.

Alzheimer’s disorder is generated by Reg := ‘frontal ‘, N_P _:= ’cortical ‘, Dir_O_^M ^:= ’’ and M_P _:= ‘degeneration’ that gives o:= O_P_^M ^ = ‘cortical degeneration’ and d = ‘frontal cortical degeneration’ because Seg := ’’, Site := ’’, e := ’’, and p := ’’. Clearly, Alzheimer’s is not (yet) a disease since the etiology is unknown (e := ’’).

Alzheimer’s disorder has a characteristic morphology, but frontal brain atrophy diagnosed by CT- and MRI-scans may have other causes. The uncertainty is accounted for by selecting the more unspecific terms Reg := ‘frontal ‘, Dir_O_^M ^:= ’a’ and M_P _:= ‘trophy’, which generates O_P_^M^ = ‘cortical atrophy’ and d = ‘frontal cortical atrophy’. The meanings of *atrophy* and *degeneration* differ.

Morphological cell line disorders are stated and recognized by formula 3) with X = L. Thus, when N_L _:= ‘myelo’, Dir_P _:= ‘dys’ and M_L _:= ‘plasia’ the disorder is O_L_^M^ = ‘myelodysplasia’.

In ICD-10 ‘Myelodysplasia’ is used for developmental disorders of the central nervous system only and does not occur as a hematological disorder [10]. This is quite unfortunate because hematologic myelodysplasia is a fairly common disorder. In this study the difference between the hematologic and neurologic myelodysplasias is clarified by using the different variables O_L_^M^/N_L_^M^ and O_P_^M^/N_P_^M^, respectively. Similar ambiguities are resolved by using the same principle.

Cells exhibit morphological disorders M_C_ that are characterized by formula 3) with X = C and 

M_C_ = ‘surface membrane leakage’ | ‘lysis’ |’necrosis’ | ‘proliferation’ |’apoptosis’ …

If N_C _:= ‘hemo’, Dir_C_^M ^:= ‘’ and M_C _:= ‘lysis’ then O_C_^M^ = ‘hemolysis’. Slightly more complicated is N_C _:= ‘Hepatocellular ’, Dir_C_^M ^:= ‘’ and M_P _:= ‘surface membrane leakage’ which gives O_C_^M^ = ‘Hepatocellular surface membrane leakage’.

Today, cell proliferation and apoptosis are measured on many tissue samples. Disorders of these processes are accommodated for by for example N_C _:= ‘B-lymphocyte ’, Dir_C_^M ^:= ‘hypo’ and M_P _:= ‘apoptosis’, which gives O_C_^M^ = ‘B-lymphocyte hypoapoptosis’, which is observed in certain lymphomas. β-cell hyperplasia is a rare disorder, but it is encountered in clinical practice. The diagnosis is generated by N_C _:= ‘β-cell ’, Dir_C_^M ^:= ‘hyper’ and M_P _:= ‘plasia’ However, β-cells are encountered in various body parts such as the pancreas and pituitary gland. The organ term N_P _:= ‘pancreatic ’ and N_I _:= N_L _:= ’’ in O_C_^M ^:= N_P_N_I_N_L_N_C_Dir_C_^M^M_P_ eliminates the ambiguity.

The treatment of leukemia depends on the affected cell line and arrested maturation stage. Highly specific drugs are used in promyelocyte leukemia (AML-M3). The systematic name of the disorder is ‘promyelocyte neoplasia’. In order to comply with clinical praxis, we reformulate using the rule: IF N_C_ = ‘promyelocyte ’ and Dir_C_^M^ = ‘neo’ THEN Dir_C_^M ^:= ’’ and M_P _:= ‘leukemia’, which generates ‘promyelocyte leukemia’. The rule works for other leukemias and lymphomas too (not shown).

##### Function

Names of disorders of function are specified by the variable O_X_^Y^ with Y := F and X := P, X := T, X := S, X := I, X := L or X := C and formula 3). Accordingly, Dir_O_^F^ := ‘hypo’, F_P _:= ’function ‘ and ‘N_P_ := ‘gonad ‘ generates the rather unspecific diagnosis O_P_^F^ = ‘gonad hypofunction’. But such diagnoses are quite useful. *Hepatocyte hypofunction* means that the secretion of albumin and coagulation factors, and the conjugation of bilirubin and secretion of bile acids are all reduced. Thus, the diagnosis sums up a lot of information in two words. Similar diagnoses for overall function such as *pulmonary hypofunction*, *myocardial hypofunction,* and *hypogonadism* follow the same rule. Also, unspecific polymorphonuclear neutrophilic granulocyte (PMN) hypofunction is diagnosed as O_C_^F^ = ‘PMN hypofunction’ suffices in some clinical situations.

But many important disorders require higher resolution of the clinical problem at hand. Typical instances are isolated hormone deficiencies and isolated hyperbilirubinemia without other signs of liver failure. Also, the emergency discrimination between heart failure due to myocardial contraction hypofunction and heart rhythm disturbance is crucial for the selection of treatment and prognosis. Therefore, function disorders partition into genomics F_P_^G^, mechanical F_P_^M^, electric F_P_^E^, metabolic F_P_^B^, humoral regulation F_P_^H^, optic F_P_^O^, thermal F_P_^R^, immune F_P_^I^, and mental F_P_^N^.

##### Genomic diagnoses

Names involved in genome diagnoses are varied. We have to cover chromosome abnormalities, gene families [36], point mutations [37], and translocations [38]. The morphemes are:

N_G _:= ‘Chr 1’ | … | ‘Chr 46’ | ‘Philadelphia chromosome’ | ‘X0 ’ | ‘XXY ’ |’G506A ’ |’*SLC37 family *’ | ‘t(8;21)(q22;q22.1) ‘ ….

The molecular changes are defined for the actual parenchyma _P_ by.

F_P_^G^ = ’mutation ’ | ‘aneuploidy ’ | ‘translocation ’ | ….

##### Mechanical functions

Terms describing mechanical functions are:

F_P_^M ^:= ’function ina ’ |’contraction ’ | ‘relaxation ’ |’sliding ’ | ‘rolling ’ |’systole ’ | ‘diastole ’ |’stability ’ | ‘tonia ’ | ‘spasticity ’ | ‘rigidity ’ | ‘shivering ’ | ‘kinesia ’ |… ….

We limit the discussion to hypertonia. *Hypertonia* refers to motor disorders of the nervous system and divides into spasticity, dystonia, and rigidity [39]. *Hypertonia* is derived from Dir_O_^F ^:= ’hyper’, F_P_^M ^:= ’tonia’ and Dir_O_^F^F_P_^M^. *Dystonia* is expressed by Dir_O_^F ^:= ’dys’ and F_P_^M ^:= ’tonia’. The other alternatives are simply Dir_O_^F ^:= ’’ combined with F_P_^M ^:= ’spasticity’ or F_P_^M ^:= ’rigidity’. N_P_ in o := N_P_Dir_O_^F^F_P_^M^ specifies the anatomical location of the disorder.

##### Electric functions

The surface membrane of all cells has electric functions. For practical reasons, we limit the cell types to striated and smooth muscle, and nerve cells. Disorders of frequency are described by the prefixes and the words fibrillation and flutter.

F_P_^E ^:= ’function ina ’ |’frequency ’ | ‘amplitude ’ | ‘axis ’ | ‘arrhythmia ’ | ‘fibrillation ’ | ‘flutter ’ | ‘amplitude ’ | ‘synchrony ’ | ‘spike ’ | ‘epilepsy ’ ….

For example, let N_P _:= ‘atrial’. Then Dir_O_^F ^:= ‘’ and F_P_^E ^:= ‘fibrillation’ generates O_P_^E^ = ‘atrial fibrillation’. If Dir_O_^F ^:= ‘extra’ and F_P_^E ^:= ‘systole then O_P_^E^ = ‘atrial extrasystole’. Also Let N_P _:= ‘sinus ’, Dir_O_^F ^:= ‘tachy’ and F_P_^E ^:= ‘arrhythmia’ then O_P_^E^ = ‘sinus tachyarrhythmia’.

For ventricular arrhythmias N_P _:= ‘ventricular ’. Then F_P_^E ^:= ’ flutter’ generates O_P_^E^ = ‘ventricular flutter’. If Dir_O_^F ^:= ‘extra’ and F_P_^E  ^:= ‘systole then O_P_^E^ = ‘ventricular extrasystole’. Also, let N_P _:= ’ AV-node ‘, Dir_O_^F ^:= ‘brady’ and F_P_^E ^:= ‘arrhythmia’ then O_P_^E^ = ‘AV-node bradyarrhythmia’.

Next take some cerebral arrhythmias. F_P_^E ^:= ’ epilepsy’ can generate four types of epilepsy by selecting either Reg := ‘frontal’, Reg := ‘temporal’, Reg := ‘parietal’ or Reg := ‘occipital’. The diagnosis of various specific epileptic types can be differentiated by adding F_P_^E^ terms that are selected from EEG measurements (not shown) see [42].

##### Metabolism and regulators

The variable K and L in formula 3) is introduced for terms naming metabolites and regulators. The two are often related such as glucose and insulin. Also, two regulators TSH and thyroxin (T4) are closely related. The variables N_X_, F_P_^X^ and F_C_^X^ allow X = B or X = H. Therefore, K and L occur as K := Dir_B_^F^N_B_, K := ’’, L := Dir_H_^F^N_H_, and L := ’’. The terms are:

N_B _:= ‘glyc ’ | ‘glycogen ’ | ‘triglyceride ’ | ‘DNA ’ | ‘purine ’ | ‘cytosine ’ | ‘albumin ’ | ‘myosin ’ | ‘actin ’ | ‘pigment ’ | ‘melanin ’ | ‘rhodopsin ‘….

N_H _:= ‘insulin’ | ‘thyroxin’ | ‘FSH’ | ‘NGF’ | ‘TGFβ’ | ….

F_P_^B^ and F_C_^B^ determine the scope of O_P_^F^ and O_C_^F^. Overall metabolic parenchyma function is described by F_P_^B^.

F_P_^B ^:= ’function ina’ | ‘secretion’ | ‘absorption’ | ‘intracellular metabolism’ | ‘receptor activity’ | ‘membrane channel activity’ | ‘concentration’ | ….

F_P_^H ^:= ’function ina’ | ‘secretion’ | ‘receptor activity’ | ‘concentration’ | ….

In this list *glyc* is the term for *glucose* used in metabolic diagnoses such as *hyperglycemia*. Also, formula 3) generates diagnoses as varied as *adipocyte triglyceride hyposecretion* and *hematopoietic stem cell DNA synthesis*.

Since the β-cells’ major product is insulin, type I diabetes is the disorder given by N_C _:= ’β-cell ‘, N_H _:= ’insulin ‘, Dir_H_^F ^:= ’hypo’ and F_C_^B ^:= ‘secretion’ diagnosed as O_C_^H^ = ‘β-cell insulin hyposecretion’ using O_C_^F ^:= N_C_N_H_Dir_H_^F^F_C_^B^. Note that the diagnosis *diabetes type I* lacks terms for etiology and pathogenesis and is incomplete.

##### Optic

The terms are:

F_P_^O ^:= ’function ina’ |’refraction’ | ‘absorption’ | ‘transmission’ | ‘metropia’ …

The diagnosis *hypermetropia* is generated by assignments Dir_O_^F ^:= ’hyper’, F_P_^O ^:= ‘metropia’. The same goes for all refraction disorders. Color vision diagnoses are accounted for by genome and metabolic function.

##### Thermal functions

The terms are:

F_P_^R ^:= ’function ina’ |’thermia’ | ….

The most common diagnoses are *hypothermia* and *hyperthermia*.

##### Immune functions

The terms are:

F_P_^I^:= ’function ina’ |’ immuno’ | ‘tolerance’ | ….

Compliance with immunological, hematological, and certain other clinical uses of diagnoses requires the introduction of a simple rule. The first character in ’immuno’ is a space. In this context the space means that if Dir_O_^F ^:= ’hypo’ then *hypo* is switched with *deficiency* to obtain *immunodeficiency*.

##### Cell functions

A more precise diagnosis is necessary in specialties such as hematology, infectious diseases, endocrinology, and immunology. Drilling down into the depth of medical complexity requires exact names for disorders of cell function. Hence, particular functions are assigned to the variable F_C_^Z^ where Z = M, Z = B, Z = H or Z = E.

F_C_^M ^:= ‘phagocytosis’ |’killing’ |’chemotaxis’ |’adhesion’ | ‘rolling’ |’sliding’ |….

F_C_^B ^:= F_P_^B^

F_C_^H ^:= F_P_^H^

F_C_^E ^:= ’resting membrane potential’ | ‘polarization’ | ‘repolarization’ | ‘conduction’ | ‘resistance’ | ….

With regard to disorders of mechanical functions of monocytes, macrophages, dendritic cells and PMN, for example, we have O_C_^F ^= ‘PMN hypophagocytosis’ O_C_^F ^ = ‘PMN hypochemotaxis’ and O_C_^F^ = ‘monocyte hypoadhesion’.

Within the scope of F_C_^E^, Dir_O_^F^ = ’’ means the healthy electrophysiological process. Dir_O_^F ^:= ’hypo’ and Dir_O_^F ^:= ’hyper’ allow for O_C_^F^ to be *hypopolarization* and *hyperpolarization*, respectively.

##### Organelle functions

Many clinical disorders are caused by disorders of intracellular functions, for example inborn metabolic storage diseases. They can be catalogued using the functions F_O_^Y^ where Y accounts for the type of function as used above with parenchyma and cells. For example, F_O_^M ^:= ‘rolling’ |’sliding’ suffices to describe the rolling of vesicles along axon fibers and the sliding of myosin along actin in contracting striated muscle cells. The number of known combinations is huge. Therefore, only one F_O_^B^ alternative is summarized.

The cell name variable N_C_ determines the scope of the functions F_O_^Y^. A large group of disorders are characterized by N_O _:= ‘mitochondria ’, Dir_O_^F ^:= ’hypo’ and F_O_^E ^:= ’resting membrane potential’, which means O_O_^E^ = ’mitochondria hyporestingmembranepotential’. The diagnosis may perhaps seem cumbersome, but it is precise.

Appropriate diagnoses may demand the inclusion of organelles. Organelle disorders are generated from the organelle name N_O_ and structural M_O_, and functional mechanical F_O_^M^, metabolic F_O_^B^ and/or electric F_O_^E^ disorders. If the function of an organelle type is disturbed in one cell line then that association is made, e.g., lysosomal enzyme N_S_ deficiency (Dir_O_^F ^:= ’a’, F_O_^B ^:= ’concentration’) in cell N_C_ of parenchyma N_P_. In general, disordered organelle function involves a substance N_S_ and occurs within the scope of a cell type and a parenchyma N_P_. The formula o := N_P_N_C_N_O_N_S_Dir_O_^F^F_O_ deriving from 3) takes the scope of substance disorders into account.

##### Mental functions

The following are diagnostic terms pertaining to conscious mental functions covering cognition and emotions:

F_P_^N ^:= ‘thinking’| ‘cognition’ | ‘intelligence’ | ‘mnesia’ | ‘perseveration’ | ‘psychosis’ | ‘anxiety’ | ‘depression’ | ‘perseverance’ | ‘mood’ | ‘syntax’ | ‘ calculia’ | ‘algebra’ | ‘ praxia’ |’short term memory’ | ‘remembering’ | ‘tranquility’ | ‘anxiety’ | ‘sadness’ | ‘melancholy’ | ‘ osmia’ | ‘ geusia’ | ‘ acusis’ | ‘tinnitus’ | ‘vertigo’ | ‘compulsion ‘ | ‘aggression ‘ |….

Amnesia and hypermnesia are generated by F_P_^N ^:= ‘mnesia’ and O_P_^F ^:= Dir_O_^F^F_P_^N^. Alzheimer’s disorder can be given the diagnosis ‘frontal brain hypomnesia’ with N_p _:= ‘frontal brain’, Dir_O_^F ^:= ’hypo’, F_P_^N ^:= ‘mnesia’ and O_P_^F ^:= N_p_Dir_O_^F^F_P_^N^. The systematic equivalent diagnosis to Korsakoff’s psychosis is ‘bilateral hippocampal afunction’ generated by Site := ’bilateral ‘, N_p _:= ’hippocampal’, Dir_O_^F ^:= ’a’, F_P _:= ’function’. The term *praxia* may seem a little odd but is needed to generate *apraxia*.

Sensory receptors transmit to afferent nerves and the mind perceives and records the stimuli. Vertigo, for example, is defined as consciousness of disordered orientation of the body in space. Several diagnoses, e.g., amaurosis, anosmia, ageusia, parageusia, deafness, and hyperacusis are relevant to afferent neural pathways and their place in 3) is immediate.

#### Connective tissue

Connective tissue is part of the general organ. The morphology of fibroblasts, fibrocytes, and mast cells is treated like other cells. *Lung fibrosis* is equivalent to *lung fibrocyte hyperplasia and collagen hypersecretion*. The systematic equivalent to *liver cirrhosis and liver fibrosis* is *liver fibrocyte hyperplasia and collagen hypersecretion*. In a disease classification *fibrosis* is simpler than *fibrocyte hyperplasia and collagen hypersecretion* and the former can replace the latter in a classification. However, the complex term is easier to acknowledge in automated CDM.

#### Tube systems

##### Morphology

Terms describing morphological disorders of tube systems are:

M_T_ := 'stenosis’ |’ occlusion’ |’ dilatation’ |’ diverticulum’ | ‘ aneurysm’ | ‘ectasia’ | ‘ perforation’ |’ erosion’ |’ ulcer’ |’ fistula’ |’ rotation’ ….

Formula 3) with N_T_ and M_T_ give rise to well-known diagnoses such as coronary stenosis, carotid occlusion, aorta aneurysm, bronchiectasia, and gastric erosion and -perforation. With N_P _:= ‘hepatic’, N_T _:= ‘canaliculi’, Dir_T_^M ^:= ‘a’, M_D _:= ‘genesis’ we get the rare syndrome O_T_^M^ = ‘hepatic canaliculi agenesis’.

##### Function

Function disorders of tube systems are assigned to the variable

F_T _:='tension’ |’resistance’ |’flow rate’ |’containment’ |’leakage’ | ‘hemorrhage’ | ‘epistaxis’ | ‘petechia’ ….

The direction of change Dir_T_^F ^:= ’hypo’ or ’hyper’ is naturally concatenated with F_T _:= ‘tension’, which through O_T_^F ^:= Dir_T_^F^F_T_ gives ‘hypotension’ or ‘hypertension’. This assignment covers a vast number of functions in various tube systems. If N_T _:= ‘arterial ‘, Dir_T_^F^:= ‘hypo’ and F_T _:= ‘tension’ formula 3) immediately gives ‘arterial hypotension’. Naming conventions are independent of the particular organ: hypertension is hypertension whether the affected tube is in the cardiovascular system, genito-urinary tract, the bile tract or the gastrointestinal tract. These similarities across organs profoundly simplify the generation of diagnoses.

#### Slits and cavities

##### Morphology

Morphological terms that refer to concepts in a model for morphological disorders of cavities and slits are.

M_S_= 'hydro’ |’pneumo’ |’hemato’ |’pyo’ …

M_S_ generates an exception in formula 3) because O_S_^M ^:= N_S_Dir_S_^F^M_S_ and Dir_S_^F ^:= ’’ gives rise to thoraxhydro, for example, which is a misnomer. Therefore, if X = S then O_X_^M ^:= N_X_Dir_S_^F^M_X_ is automatically converted to O_S_^M ^:= M_S_N_S_. The modified formula generates diagnoses such as *hydrothorax, pneumothorax, hematopericard* and *pyonephrosis*.

##### Function

Some of the terms are:

F_S_:= 'compliance’ |’pressure’ |’ restrictive’ | ….

The peritoneal and pleural slits allow smooth movement of its organs, bowels, abdominal wall, and diaphragm against each other. The terms allow diagnoses such as restrictive pericarditis.

### The etiology variable e

The classes beneath the root node of the etiology hierarchy are shown in Table 1. They provide the roughest etiology terms that can be assigned to e. The leaf nodes are individual etiological agents like particular mutations and infectious agents. Terms located between the first-level node and the leaf nodes have an intermediary specificity such as *gram-negative bacteria*. The etiology influences the choice of suffix S_E_ that modifies the names N_X_ of body parts, tissues, and cells in 3).

**Table 1:** Terms and the variable e used in the etiology segment of diagnoses. S_E_ is the suffix variable

**e**	**S**_E_
‘Prothrombin *G20210A* mutation’ …	‘pathy’, ‘osis’
‘Trauma’ | ‘Car accident’ | ‘fall’ | ‘knife stab’ | ‘bite’ …	‘ vulnus’ | ‘ fracture’ …
Substance name	‘ intake’
‘Infectious’ | micro-organism name	‘itis’
‘Allergic’ | allergen name	‘itis’
‘Thermal’ | ‘frost’ | ‘burn ‘…	‘itis’
‘Radiation’	‘osis’
‘Social’ | social problem name	‘pathy’
‘Essential’’ | ‘idiopathic ‘	‘’ | ‘osis’

Formula 2) allows the diagnoses *viral myocarditis* and *Coxachie virus B1 myocarditis* depending on the diagnostic accuracy. The suffix ‘itis’ signifies the presence of a microorganism or an allergen in the primary affected body part. For example, with formula 2) and e := *’Staphylococcus aureus*’, N_p _:= ’ myocard’ and S_E _:= ’itis’ we get d:= ’*Staphylococcus aureus* myocarditis’, which means presence of the bacteria in the myocardium, in accordance with the clinical model. The principle obviously extends to hereditary-, allergic- and radiation etiology.

Nutrients and vitamins slightly complicate matters. The intake of a substance may be too high (elevated) or too low (diminished) such that

$$\text{Dir}_\mathrm{E}=^{\prime}\text{Elevated}^{\prime}\,|\,^{\prime}\text{Diminished}^{\prime}$$  

In the context of nutrition e := Dir_E_^F^&N_B_&’intake’. Thus, if N_B _:= ’glucose’ and Dir_E _:= ‘Elevated ‘ then e = ‘Elevated glucose intake’. The formula easily discriminates the unspecific *Elevated sugar intake* from the specific *Elevated glucose intake* and applies equally well to vitamins and other substances. The affected organ, tissue and cells are modified to N_P_&S_E_, N_I_&S_E_ and N_C_&S_E_, respectively, where S_E _:= ’osis’ (Table 1).

Complex sociopsychosomatic disorders can also be expressed by formula 2). Let e := ‘Social ‘, N_p _:= ‘*neur*’, p :=‘‘, S_E _:= ‘*osis*’. Then 3) generates the unspecific diagnosis *Social neurosis*. These etiologies accurately describe clinical facts and are amenable to automated generation of the etiologic part of diagnoses.

### The pathogenesis

The pathogenesis links a primary affected body part (source) with some secondary affected body part (targets). The link and the target together are abbreviated by p. The source of the link derives from parenchyma o. The pathogenesis may be one-way from source to target. Typical examples are emboli and metastases. Alternatively, there is a two-way link from source to target and back to the source. The source and target may be the same or differ. If they are the same then the pathogenesis only describes the link. Otherwise, the pathogenesis contains the term of the target too. These properties are determined by the actual context. Terms used with p are summarized in Table 2.

**Table 2:** Terms used to describe pathogenetic mechanisms. The variables are p and the suffix S_P_. The sign ‘|’ denotes alternatives

**Pathogenesis**	**p**	**S**_P_
**Connective tissue**		
Intercellular space	‘edema’ | ‘dehydration’ | …	‘’
	’sclerosis’ …	‘osis’
**Hemostasis**	‘hemat’ | ‘thrombosis’ |’bleeding’ | ‘petechia’| ‘hematoma’| ‘hemorrhage ‘…	‘oma’
**Circulatory system**		
	‘inflammation’ | ‘ischemia’ |’infarction’	‘ ‘ | ‘ial’ | ‘al’
**Immune reactions**		
Type 1	‘IR1’	‘itis’
Type 2	‘IR2’	‘itis’
Type 3	‘IR3’	‘itis’
Type 4	‘IR4’	‘itis’
Type 5	‘IR5’ | ‘granuloma’	‘itis’
**Intercellular metabolism**		
Metabolite name		‘osis’
Precipitates, stones		‘lithiasis’
**Humoral regulators**		
Regulator name		‘‘ | ‘osis’
** Nervous system**		
Afferent Efferent	‘ psychosomatic’ ’ somatopsychic’	‘osis’
Transmitter	‘ hyper N_H_ secretion’|’ hypo N_H_ ‘ secretion’|’ ‘ hyper N_H_ reabsorption’| ‘ hypo N_H_ reabsorption’|…	

As shown in 3) and Table 2 the suffix S_P_ modifies names of organs, tissues, and cells in the same way as S_E_ (Table 1). In most cases S_E_ = S_P_. However, it might occur that S_E_ ≠ S_P_. Then, as seen by comparing Table 1 with Table 2, the etiology conflicts with the pathogenesis. The conflict has to be resolved by returning back to CDM and possibly dividing the clinical problem at hand on two or more different diagnoses.

For the localization of p we only need the suffix variable L_H_. Fortunately, several suffixes and terms are already in use.

L_H _:= ‘aemia’ | ‘uria’ | ‘oma’ | ‘myelia’ | ‘ suggulation’ | ‘emesis’ ….

Note that the variable L in 3) is within the scope of disorder, while L_H_ is within the scope of the pathogenesis. The scope precludes the confusion of L with L_H_.

#### Extracellular space (ECS)

In *lung fibrosis* the fibrosis is considered to be the source o := N_P_N_I_S_P_ where N_P _:= ’lung’, N_I _:= ’fibr’ and S_P _:= ’osis’. We know that lung fibrosis causes lung hypofunction. Thus, we have the same source and target organ and N_P _ = ’lung’ in the generation of o and p. The partial diagnosis p := ‘ causing ‘ & ‘lung hypofunction’ is generated by p := ’causing ‘ & N_P_Dir_O_F_P_ where Dir_O _:= ’hypo’ and F_P _:= ’function’. These assignments complete the partial diagnosis op = ’lung fibrosis causing lung hypofunction’ as required.

Sclerosis means that calcium is deposited on extracellular fibers or precipitated with extracellular cholesterol and other negatively charged lipids. The composite term *sclerosis* could have been generated by N_C _:= ‘scler’ and S_P _:= ‘osis’, but sclerosis is a composite material and not a cell. Therefore, *scler* cannot be assigned to N_C_. The assignments p := ‘scler’ and S_P _:= ‘osis’ would require the concatenation of a pathogenetic mechanism with a suffix (pS_P_) that violates the formula 3). Therefore, we choose p := ’sclerosis’ (Table 2) and generate *arteriosclerosis* by o := N_T _:= ’arterio’ & op.

Precipitates are marked by the suffix lithiasis. As with sclerosis, their localization is given by op. *Urolithiasis* means a stone somewhere in the urinary tract. *Cholecystolithiasis, nephrolithiasis* and *phlebolithiasis* constrain the location to the gall bladder, the kidneys and veins, respectively, and are generated as shown above.

Edema and dehydration are common disorders of ECS. For *edema* we choose the appropriate location in 3) p := ’ edema‘. With Reg := ’conjunctival’ we derive *conjunctival edema*. The cause of the edema, e.g., renal failure, is given by o.

#### The vascular and lymphatic circulation

Terms that can be assigned to the variables p and S_p_ are found in Table 2. For example, the unspecific diagnoses *myocardial infarction* and *cerebral ischemia* are generated from op where o = N_P_. The terms are selected during CDM. Also, emboli occlude arteries, so we may have M_T _:= ’occlusion’, but this is excessive.

CDM may lead to the unspecific cerebral ischemia (transient ischemic attack (TIA)) or infarction, or the more specific occlusion of a particular cerebral artery such as *left medial cerebral artery embolus*. I prefer the latter, which is obtained by N_T _:= ’left medial cerebral artery’. In the present example, O_T_^M ^:= SiteN_T_M_T_, i.e., O_T_^M^ = ’left medial cerebral artery occlusion’. Formula 5) requests both the morphology and the affected function(s), which in the present example are linguistic and motor functions. Here, O^F ^:= ’aphasia and right hemiparesis’ so formula 4) gives *left medial cerebral artery occlusion causing aphasia and right hemiparesis*.

##### Metastasis

Metastasis is readily described by for example o := ’colon carcinoma’ and p := ’with ‘& RegSiteN_P_O_P_^M^O_T_^M^O_S_^M^O_I_^M^ ' metastasis’. When one site is chosen the others are set empty. For example, if N_P _:= ’pulmonary’ then Reg = Site = O_T_^M^ = O_S_^M^ = O_I_^M^ = ’’. This instantiates op as *colon carcinoma with pulmonary metastasis*.

##### Embolism

Emboli originate as thrombi at the source site and become stuck at the distant target site. Here we have o := N_T_ & ‘ thrombosis’ and p := ’ with embolus to ‘N_P_ ‘ and ‘N_P_ & ‘ infarction’, which among others translates into *deep vein thrombosis with embolus to lung and lung infarction*.

The variable p depends on the link, i.e., the vascular and lymphatic path that determine the target. If the source in o is N_S _:= ’left heart auricle’ the path and the target is given by p = ’ embolus to’ & N_T_ and the affected N_P_ is uniquely determined by N_T_. Alternatively (and rarely), if the source is not the heart then it is a vein, or the venous plexus of the pelvis and the path is through the foramen ovale. In that case, p = ’ embolus from ’ & N_T_ ‘ through foramen ovale’. The term *heart* discriminates between the two alternatives.

##### Bacteremia, viremia, fungemia and septicemia

The source is always the infected body part described by the variables e and o and L_H _:= ’emia’. In bacteremia, viremia and fungemia the microorganisms spread with the blood stream but do not harm secondary affected organs. Thus, p := eL_H_, for example *streptococcemia* and *nocardemia.*

Septicemia, sepsis syndrome, septic shock and systemic inflammatory response syndrome (SIRS) are unclear clinical notions. Precision is vastly increased by accurate description in the variable o, which gives the primary affected body site and its altered function(s). Then, the secondary affected body sites and their hypofunctions are clearly defined in the same way as *lung fibrosis* above. We are then able to describe the assumed cause of tachycardia and hypotension as required for septic shock. Note that the duration and prognosis are derived parameters, and not assumed in the sepsis diagnoses. The pathogenetic formulas eliminate ambiguities inherent in existing diagnoses describing the spread of microorganisms.

#### Intercellular metabolism

Some conventional diagnoses are constructed from the names of organs, cells, etiology, and pathogenesis, but most such diagnoses are incomplete. The diagnosis *hyperglycemia*, for example, leaves open alternative pathogenetic mechanisms like *hypoinsulinenia, hypercortisolemia* and *hypersomatotropinemia*, and a series of other pathogenetic mechanisms. Again, hypoinsulinenia can be caused by disorders like insulin hyposecretion and insulin resistance. In addition, the diagnosis *hyperglycemia* ignores etiological causes such as autoimmune reactions against β-cells, alimentary hyperglycemia, chemical etiology (alcohol-induced pancreatitis) and hereditary glucose channel mutations. Accordingly, even seemingly structured conventional diagnoses often lack the explanatory power that is necessary for adequate therapy and follow up.

Let the variable N_B_ hold the names of substances and metabolites. The concentration of a substance N_B_ is measured at localization L_H_ and described by p := N_B_L_H_, which generates for example *glucosuria*. *Hyperglycaemia* is derived from p := Dir_P_N_B_L_H_ and the appropriate instantiation of the variables. The formula readily generalizes to other metabolites (fatty acids, enzymes, electrolytes, etc.).

There is a close relation between metabolic disorders O_P_^A^ and O_C_^A^, and metabolic pathogenesis p. The name of the secreted and absorbed substances N_B_ is the same as that measured in peripheral blood. We saw above that O_C_^F ^:= N_C_N_H_Dir_H_^F^F_C_^H^ can describe type I diabetes and made the assignment o := O_C_^F^. From above we have p := Dir_P_N_B_L_H_ that is modified to p := ‘ causing ‘ & Dir_P_N_B_L_H_ in the present context. In general, op describes the causal relations between cell disorders and observed blood concentrations, for example *hepatocyte* *albumin hypoproduction with albumin hyposecretion causing* *hypoalbuminemia* and *hepatocyte* *bilirubin hypoconjugation causing* *hyperbilirubinemia*.

Formula 3) relates the etiology to organ failure and the pathogenesis and incorporates etiological and pathogenetic explanations. It also supplies a bridge between diseased systems and the names that describe the disease. Accordingly, diagnoses constructed by formula 3) and its extensions are likely to be complete.

#### Immune reactions (IR)

Immune reactions are denoted by concatenating organ names with the suffix S_P_ = ’itis'. Thus, thyroiditis is an IR of the thyroid. Its cause depends on the etiology (microbial, allergic, or hereditary (autoimmune)) obtained from the variable e. A variety of diagnoses are derived from formula 3) simply by d:= e&o&p where S_E_ = S_P_, e is an appropriate etiologic agent, o := N_X_S_P_ and p drives from Table 2. The variable p makes the IR type explicit such as with p = ’ IR4’. Formula 3) can be extended with morphology and functions as described above.

S_E_ = S_P_ with e =’’ or p = ’’ or both leaves ambiguous diagnoses. For example, the diagnosis *hepatitis* means different things in different contexts that have different therapeutic and prognostic consequences. For example, *Viral* *hepatitis B* and *Viral hepatitis C* requires different treatment and follow up than *autoimmune hepatitis*. Likewise, *conjunctivitis* *IR1* is very different from *conjunctivitis IR3*. The former probably has an allergic etiology whereas pyogenic bacteria usually cause the latter. Therefore, clinicians should always aim for complete diagnoses with e ≠ ’’, o ≠ ’’ and p ≠ ’’. In fact, the diagnosis is deficient whenever the suffix ‘itis’ is not followed by the type of immunological reaction: either colitis IR3 (ulcerating colitis) or colitis type IR4 and IR5 (Krohn’s disease). Soon, it might be possible to pass names N_H_ of disorders of interleukins, cytokines, etc. into IR diagnosis, which is easily realized with formula 3).

#### Hemostasis

Bleeding from the stomach and kidneys manifest themselves as *hematemesis* and *hematuria*, respectively. In this context we choose p := ’hemat’ and generate the *hematemesis* and *hematuria* recursively by p := pL_H_. *Hematemesis* is generated by L_H _:= ’emesis’ and *hematuria* by L_H _:= ’uria’. Reg and Site are used to localize suggulations and hematomas.

In thrombosis the source and target are in the same organ. Two common examples are o := N_T_ ‘ stenosis’ p = ’causing ‘N_P_ ‘ ischemia’ and o := N_T_ ‘ thrombosis’ p = ’causing ‘N_P_ ‘ infarction’ that are instantiated to *coronary stenosis causing myocardial ischemia* and *coronary thrombosis causing myocardial infarction*.

#### Neural pathways

The nervous system links together social events, the brain, and the mind to other organ systems. These links allow sociopsychosomatic and somatopsychosocial diseases and disorders, respectively.

##### Psychosomatic

Complex psychosomatic and somatopsychic diseases are expressed by formula 3) with e := ‘Social ‘, N_p _:= ‘neur’, p := ‘‘, S_P _:= ‘osis’, which by d:= eoS_P_p becomes *social neurosis*. The etiology e = ‘social ‘ implies that the cause of neurosis is due to some social problem, and not the consequence of a mental disease with another etiology such as heredity or drugs. The assignment formalizes the notion of sociopsychosomatic disease and fits the biopsychosocial clinical model (CM) [35].

The general formula for psychosomatic disorders is op where o := O_P_^F^ is the function of some part of the brain or mind and p := EffO_X_^F^. The variable Eff types the disorder as mediated by a named efferent part of the nervous system and O_X_^F^ is the secondary affected organ. For example, Eff := ‘ N. X’ describes the involvement of the vagus nerve and O_P_^F ^:= ’bradycardia’. The diagnosis could be simplified to p = ’ causing ‘O_X_^F^ (see above) and adding a path suggests a therapeutic option.

Naming the social problem specifies the etiology (Table 1). *Hyperventilation syndrome* is a paradigmatic example of a psychosomatic disorder with unknown etiology but does not tell whether it is a primary or secondary disorder. Table 1 identifies possible etiologies. Also, the pseudoalgorithm generates possible differential diagnoses SegO_P_^M^ = ’pontine tumor’ and SegeoS_E_ = ‘pontine viral encephalitis’.

Anxiety and panic disorders are other debilitating disorders with varied etiology and pathogenesis. Several brain regions are involved. For example, amygdala may be the primary affected body part and p may involve tachycardia, hyperventilation, etc. as described above.

##### Somatopsychic

The constant assigned to the variable o designates the disorder in the primary affected body part. In somatopsychic disorder information is passed along some afferent nerve (Aff) and is made conscious in short-term memory O_P_^F^. Accordingly, we have p := ‘ causing ‘AffO_P_^F^. Then op may describe situations such as *gastric adenocarcinoma causing epigastric* *pain* but recall that epigastric pain is a symptom and not part of UDS.

### Etiology and anatomical localization

Epstein-Barr virus infects B-lymphocytes and spreads rapidly to many organs and tissues [40]. Therefore, infectious mononucleosis involves many anatomical sites. The systematic diagnosis is d = ‘Epstein Barr virus B-cell hyperplasia’. The same principle applies to other widely distributed cell types.

### Heterogenous etiology

Immune deficiencies are the ultimate cause of many infections. A prominent example is AIDS. Other relapsing infections are due to granulocyte disorders. A relatively rare disorder is mutation A392G neutrophil hypofunction (Diagnosis 1) involved in *Staphylococcus aureus meningitis IR3* (diagnosis 2) and *Staphylococcus aureus carditis IR3* (diagnosis 3). The general syntax for these diagnoses is: Diagnosis 1 causes diagnosis 2 and diagnosis 3. Each of the diagnoses is generated by formula 3).

### Examples of use of UDS

Three clinical examples of combinations of e, o, and p are compared with diagnosis used in medical practice (Table 3). Gluten allergy mainly affects the small intestine and that is focused here. X-ray exposure induces bone marrow hypoplasia. X-rays also affect rapidly proliferating cells including intestinal epithelium and other tissues (not shown). The etiology of distorted body image will in many cases remain unknown and the etiology of these cases remains idiopathic. As described above one diagnoses (diagnosis 1) may cause another diagnosis (diagnosis 2) which is illustrated by the complex disorder anorexia nervosa. Malnutrition reduces intestinal uptake of numerous nutritional elements as reflected in the pathogenesis.

**Table 3:** Relationships between e, o, and p and commonly used clinical diagnoses

e	o	p	common clinical diagnosis
Gluten allergy	Small intestinal mucosal atrophy with malabsorption	IR2a	Gluten induced celiac disease
1.idiopathic 2.malnutrition	1.Distorted body image 2.Adipose tissue and striated muscle atrophy	1.psychosomatic 2.hypoglycemia, hypokalemia, …	anorexia, bulimia nervosa
x-ray exposure	bone marrow hypoplasia	fibrosis	Radiation induced bone marrow failure

^a^Immune reaction type 2 characterized by gluten antibodies

The conversion of three typical disease names to UDS is shown in Table 4. The etiology of Wilson’s disease is *ATP7B* gene mutation that primarily reduces the secretion of copper with concomitant hepatocyte damage and hypofunction. This disorder causes cupper precipitates in the iris edge and lenticular nucleus degeneration and hypofunction. Through neural pathways the latter affects the function of many body parts.

**Table 4:** Conversion of typical disease names to UDS

Typical disease name	e	o	p
Wilson’s disease	*ATP7B* gene mutation	1.hepatocyte lysis, hypoplasia and hyposecretion 2.iris edge 3.lenticular nucleus degeneration and hypofunction	1.IR4, cirrhosis^a^, metabolic pathogenesis (hypoceruloplasminemia, hypoalbuminemia, hyperbilirubinemia, … 2.Copper precipitate 3.Neural pathways
Huntington's disease	dominant huntingtin gene hyperconcatenation of cytosine-adenine-guanine (CAG) repeats	1.striatal degeneration and atrophy 2.hypocognition, 3.psychosis, compulsions, aggression,… 4.Huntingtin with > 36 glutamine residues	1.cortical brain-derived neurotrophic factor (BDNF) depletion 2a. dyskinesia 2b. late behavioral hypoactivity
multiple sclerosis	1.essential 2.Epstein-Barr virus	Central nervous demyelination and nerve cell hypofunction	IR4^a^

^a^Immune reaction type 4 characterized by lymphocyte infiltration

The hereditary etiology of Huntington’s disease is well known. As shown in Table 4 the disorder manifests itself morphologically, and by motor, cognitive, emotional metabolic disturbances. Depletion of BDNF [41] and altered behaviour are examples of regulator and psychosomatic pathogenesis, respectively.

The etiology of multiple sclerosis often remains idiopathic, but in recent years Epstein-Barr virus is assumed to cause damage oligodendrocytes causing demyelination. In clinical situations the discrimination between idiopathic and Epstein-Barr virus etiology has to be made on an individual patient basis. The disorder is worsened by IR4.

### Extensions to UDS

Formula 3) is sufficient for many purposes but complete diagnoses describe the time course (Dur), prognosis (Prog) and degree (Deg). This naturally accomplished by extending formula 3) to

6 $$d:=\mathrm{DurSegRegSiteProgeoSpDeg}$$

### Time course

Time course is specified by the following alternatives:

Dur := ‘*peracute* ‘ |’*acute* ‘ |’*subacute* ‘ |’*chronic* ‘ |’*chronic remittent* ‘ |’*chronic progressive* ‘| ‘*paroxystic ‘ |….*that explicitly state the rapidity of onset and duration of diseases and disorders. The naming convention places time course to the far left in diagnoses as seen with example *acute appendicitis* and *chronic hepatitis* as in 6). Terms such as chronic, chronic remittent and chronic progressive are used with liver diseases.

### Prognosis

The prognostic terms *benign* and *malignant* can stem from pathology or survival. The former is closely related to morphological changes such as a malignant neuroblastoma, whereas the latter is determined by clinical observations. UDS avoids the pathological-anatomical use of malignant. The term neoplasia and anaplasia are preferred. Accordingly, the terms benign and malignant are reserved for survival. This use eliminates an ambiguity that plagues many textbooks and scientific articles. Prognosis is described by the variable

Prog := ‘benign ‘ | ‘semi-malignant ‘ | ‘malignant ‘ ….

With Prog := ‘*malignant* ‘, e = ‘*hereditary* ‘, o := ‘*thyroid neoplasm*’, Suff := ‘’ and p := ’’ the diagnosis reads d = ‘malignant hereditary thyroid neoplasm’. Likewise, the formula can generate d = ‘malignant hyperthermia’.

### Degree—severity

Some classifications allow for the concept of degree [42]. Degree is common to many clinical scales such as grading the level of functioning [43], the severity of heart failure [44] and liver function [45–47]. Highly differentiated, little differentiated and undifferentiated tumor that justifies the diagnosis are placed in the pathologists’ narratives. At the level of diagnoses verbal expressions of the degree of differentiation are better replaced with a systematic degree in the range 0–5 as practiced in cytology (see below).

Terms such as *preleukemia*, *subclinical celiac disease*, *subclinical hypothyroidism*, and p*reclinical*-, *subclinical*- and *latent diabetes mellitus* are frequently encountered in medical textbooks. The concepts *preclinical*, *subclinical,* and *latent* imply a disorder without clinical manifestations. They describe similar states, and all can be substituted by degree 0, which announces abnormal supplementary observations but no symptoms and signs. Manifest disease implies the presence of symptoms or signs. Manifest disease is often graded in the four degrees {1, 2, 3, 4}. Deg is the variable denoting the degree:

$$\mathrm{Deg}:=\mathrm{^{\prime}}degree \ 0\mathrm{^{\prime}} |\mathrm{^{\prime}}degree \ 1\mathrm{^{\prime}} |\mathrm{^{\prime}}degree \ 2\mathrm{^{\prime}} |\mathrm{^{\prime}}degree \ 3\mathrm{^{\prime}} |\mathrm{^{\prime}}degree \  4\mathrm{^{\prime}}$$

Typical examples that illustrate the variety of generated diagnoses are *cardiac insufficiency degree 3*, *hypertension degree 4*, *cervical squamous cell dysplasia degree 4* and *astrocytoma degree 3*.

One might object that the concepts *preclinical*, *subclinical,* and *latent* have a subtler meaning. But this information is already covered by the terms assigned to the time course, etiology, disorder, and pathogenesis.

Time course, prognosis and degree are interrelated. A fifth number in ICD-10 can be used to describe the progression of the disease from continual to episodic with a progressive deficit, episodic with a stable deficit, episodic remittent, incomplete remission, other and uncertain prognosis. Not surprisingly, the purpose of such a mixture of degree, change in degree and time course has been questioned [48]. I have not encountered a medical classification that makes the connections explicit as is done in formula 6).

## Discussion

d:= e&o&p is the first formula that generates systematic diagnoses. It rests on standard international clinical concepts and language. The formula and its extensions generates diagnoses for all medical specialties, whereby it counts as a universal medical diagnosis syntax (UDS). UDS may have implications for medical education and classifications and may create a foundation for structured clinical decision-making. Formulae are the hallmark of the hard sciences. Therefore, d:= e&o&p moves clinical medicine into the domain of hard science.

### UDS is open ended

The boost of medical knowledge challenges medical classifications and poses serious problems for medical education and CDM. The information explosion often leads to the re-evaluation of the etiology, disorders of structure and function of body parts, and the pathogenesis of known diseases. UDS opens for reasoning about the structure and content of diagnoses and predicts new diseases and syndromes. The ICD10, ICPC-2, and DSM V contain historical and some outdated diagnoses. UDS does not offer preformed diagnoses; diagnoses depends on actual knowledge and actual patients.

Disease is said to mean so many different things that it cannot be applied to psychiatry [49]. However, formula 6) adapts well to psychiatric diagnoses. The complexity of 6) with extension explains why diagnosis seems to be such a heterogeneous concept.

The present work shows that complete systematic diagnoses can be constructed from the names of organs, cells, etiology, organ disorder and pathogenesis according to formula 6). The diagnosis formulae covers social, psychological, and biological aspects of diagnoses. In spite of its wide applicability, it has few variables. The few variables make the formula easy to easy to use, control, and implement. The extended formula may also facilitate communication and reinforce agreement among physicians.

### Excluded from UDS

The development of generative grammar in the nineteen seventies and eighties distinguished logical form from grammatical form. The logical form provided the syntactic component without the aspect of meaning of sentences ([1], p.23). Montague disagreed and claimed that there is no important difference between natural languages and the artificial language of logicians ([1], p.23). In UDS formula 6) defines the syntax. The categories delimit the content of the variables. Nothing outside a valid form and appropriate terms are allowed. Syntactic errors and category mistakes are easy to spot. For example, the diagnosis *pathenceph* cannot be generated, and *Staphylococcus aureus* is not a tissue. In contrast, medical terminologies sometimes does not distinguish between assertions and pseudoassertions. UDS excludes misleading diagnoses, but to see this requires interpretation into the semantics of a clinical mode [35].

#### Clinical findings

Clinical findings consist of symptoms, signs, and supplementary investigations. They are used to ground diagnoses during medical decision-making. The ICPC-2 allows clinical findings to serve as diagnoses [11]. However, EPR can separate diagnoses and clinical findings. The latter can be extracted from clinical narratives and laboratory files and are used in automated medical decision-making [30, 33, 50, 51]. Clinical findings are suited for justification of diagnoses but not as diagnoses. None of the formulas in UDS have a symptom variable. ‘algia’ in fibromyalgia and myalgic encephalopathy (ME) refers to a symptom. Accordingly, fibromyalgia and ME cannot be derived in UDS.

#### Misnomers

A clear understanding of clinical terminology requires the elimination of misnomers. *Dysfunction* is not a misnomer. It is much used as a crude diagnosis such as *gastric dysfunction*, *dysphagia,* and *dyspepsia*. *Dysphagia* may refer to a collection of symptoms such as regurgitation and/or pain and/or …. However, such diagnoses disregard the specificity of clinical functions. The cure is to eliminate all terms where ‘dys’ is associated with clinical findings.

Obvious misnomers cannot be generated by 6). Itiscys is incomprehensible but cystitis is immediately understood. d:= eoSp allows ‘cyst’ & ‘itis’ while ‘itis’ & ‘cyst’ cannot be generated. Formula 6) constrains the possible term combinations to those derivable in school medicine.

In EPR the diagnoses are stored separately from the clinical findings. Therefore, formula 6) can be compared with the justification, which consists of the clinical criteria [30]. This procedure allows systematic check of the accuracy and precision of diagnoses. In addition, any arbitrary diagnosis will not pass the constraints imposed by d:= eoSp. For example, the diagnosis ‘hemiplegia due to coronary artery occlusion’ fails because the knowledgebase rests on a clinical model (see below), but ‘hemiplegia due to right medial cerebral artery occlusion’ will pass. The formula combined with the clinical model limits well-formed diagnoses to those that both fit clinical observations and empirical medical knowledge.

### Analysis and parsing

In a previous article, we used diagnoses to set up a semantic analysis of clinical narratives [30]. We assumed the semantics to result from parsing. Analysis divides sentences into words and word groups according to their syntactic function. In UDS analysis divides diagnoses into terms and groups them according to their place in diagnoses. Parsing categorizes individual terms in diagnoses. The categories of formula 6) are uniquely determined by their place in the diagnoses. Thus, the present work shows that UDS may fuse analysis and parsing into one process.

### Standards of acceptability

The present method requires independent assignment of strings to the variables e, o, and p. This might theoretically seem to be an impossible task in clinical practice. However, several studies suggest the contrary. First, the formula d:= eop works in hematology and infectious medicine [52]. Second, the clinical findings corresponding to the suffix ‘itis', and terms pertaining to selected organs can be obtained by automatic parsing of clinical narratives from EPR [30]. Third, names of entities pertaining to e, o and p can be automatically parsed from diagnoses in free text form [30, 51]. Preliminary data also suggest that formula 3) is useful for the analysis of social problems [33]. Finally, a combinatorial classification based on d:= eop was successfully built into an EPR [31, 35]. Taken together, these results indicate that the independent assignment of strings to e, o and p is feasible, but further independent studies are required.

### Limitations and disadvantages of UDS

This study is limited to a syntax generating diagnoses. Comments to clinical and laboratory work are limited to examples (Tables 3 and 4 with comments). This study does not comment on the semantics of UDS, i.e., does not interpret variables, terms, and connectives into a model. Aspects of semantics and models have been described [35, 53]. Further work on models of health and disease is in progress. As a result, UDS may be interpreted into the model of disease. Diagnoses are goals in CDM that may be generated by UDS, but CDM also involves clinical findings and together they are too voluminous to be treated here.

UDS may be a cumbersome disease classification in EPR. Existing implementations of ICPC-2 and ICD10 are more effective and labor-saving. However, the number of entries in ICPC-2 and ICD10 are limited, many rare disorders are not covered, and it is laborious to maintain them. A combination of UDS with ICPC-2 and ICD10 may solve these problem, but that remains to be shown. Finally, UDS may be hidden in a decision support system that suggests diagnoses.

## Conclusions

This study generates systematic diagnoses using standard medical concepts, language, and the syntax used in the classifications ICPC-2 and ICD10. The formula d:= eop with extensions give rise to universal medical diagnoses. The generation of diagnoses allows the diagnoses to be tailored to the medical problem at hand and relieves physicians from squeezing the patients’ clinical findings into some classification. In addition, novel combinations of terms may predict novel diseases.

Ambiguous diagnosis, clinical findings and misnomers that may lead to confusion and disagreement are excluded from UDS. Leibniz’s dream of a symbolism for all human thought implies that arguments could be resolved by calculation ([54] p.94). The results presented here suggest that d:= eop may have such a clinical role.

## Data Availability

NA.
